# The impact of obesity and body weight on the outcome of patients with relapsed/refractory large B-cell lymphoma treated with axicabtagene ciloleucel

**DOI:** 10.1038/s41408-021-00515-2

**Published:** 2021-07-01

**Authors:** Kitsada Wudhikarn, Radhika Bansal, Arushi Khurana, Matthew A. Hathcock, N. Nora Bennani, Jonas Paludo, Jose C. Villasboas, Yucai Wang, Patrick B. Johnston, Stephen M. Ansell, Yi Lin

**Affiliations:** 1 Division of Hematology, Department of Medicine, Mayo Clinic, Rochester, MN USA; 2 Division of Hematology and Research Unit in Translational Hematology, Faculty of Medicine, Chulalongkorn University, Bangkok, Thailand

**Keywords:** B-cell lymphoma, Immunotherapy

**Dear Editor,**

CD19 chimeric antigen receptor (CAR) T-cell therapy has revolutionized the treatment armamentarium of relapsed/refractory aggressive B-cell non-Hodgkin lymphoma (NHL) with an unprecedented response rate and potential for durable disease remission in these difficult-to-treat patients [1–3]. Obesity is a public health concern in the United States with an age-adjusted prevalence at 42.4% among U.S. adults in 2017–2018 [4]. Obesity can impact cancer therapy and outcomes in several different aspects [5]. Several studies have demonstrated obesity and body weight as important factors that influence chemotherapy dosing patterns in clinical practice. Chemotherapy dose reduction, non-weight-based, or capped dosing strategies are common practice among oncologists prescribing treatment for overweight or obese patients, including conditioning regimens in those undergoing hematopoietic stem cell transplantation (HSCT). Such approaches may result in inferior treatment outcomes in certain cancer types [6]. In contrast, accurate weight-based chemotherapy dosing was not associated with increased toxicity in obese patients [6]. In addition to its impact on dosing pattern, obesity is associated with a pro-inflammatory state, endothelial injury and immune dysregulation in pre-clinical studies [7]. Data from allogeneic HSCT indicated that obesity was associated with higher incidence of acute graft versus host disease, higher transplant-related mortality and worse survival after allogeneic HSCT [8]. In contrast, obesity may be associated with improved survival in patients treated with immune checkpoint blockade (ICB) therapy possibly due to its proinflammatory effect on immune response [9]. The effect of obesity on the pattern of lymphodepleting (LD) chemotherapy dosing, CAR T-cell delivery, immune-mediated toxicities, and outcomes after CD19 CAR T-cell therapies has not been described. Herein, we described the impact of obesity and body weight on CAR T efficacy and toxicity in patients with NHL treated with axicabtagene ciloleucel (axi-cel) at our institution.

This study was reviewed and approved by the Mayo Clinic institutional review board committee. We conducted a retrospective study including 78 consecutive patients with NHL who received axi-cel at Mayo Clinic between June 2016 and October 2020. Obesity was defined as having a body mass index (BMI) ≥ 30 using measurement at the time of LD chemotherapy. We analyzed baseline clinical characteristics, patterns of LD chemotherapy dose, CAR T associated toxicities, response to CAR T-cell therapy, and survival outcomes between obese and non-obese patients. Dosing of LD chemotherapy including its correlation with BMI were described as total dose per m^2^ of body surface area (BSA) and actual delivered dose to standard dose ratio (the standard total dose per BSA of fludarabine; [Flu] and cyclophosphamide [Cyc] are 1500 and 150 mg/m^2^, respectively, as described in previous reports [2]. The cutoff date for data analysis was January 31st, 2021. All statistical analyses were performed using R software, version 4.3.0 (R Foundation for Statistical Computing). A *P-*value < 0.05 was considered statistically significant.

Of the 78 patients, 22 (28%) and 19 (24%) patients were defined as overweight (BMI 25-29.99 kg/m^2^) and obese (BMI ≥ 30 kg/m^2^), respectively (Supplement Fig. S1). There were no statistical differences in demographic information, and disease characteristics of patients in the obesity compared to the non-obesity group except the total delivered Cyc dose per BSA (Table 1). The median total Cyc and Flu dose per BSA was 1507 (1021–1660) and 89.1 (0–104.8) mg/m^2^, respectively. Obese patients received lower Cyc dose per BSA (1501 mg/m^2^; range 1077–1525 mg/m^2^) than non-obese patients (1512 mg/m^2^, range 1021–1660 mg/m^2^) (*P* = 0.008), however, the difference was not clinically significant. We observed similar lymphodepletion effect between groups as demonstrated by comparable absolute lymphocyte count at the day of CAR T-cell infusion. Fludarabine was not given for LD chemotherapy in 1 non-obese patient with advanced age and impaired renal function on expanded access protocol (estimated glomerular filtration rate of 21 mL/min/BSA). There was a non-statistically significant inverse correlation between delivered LD chemotherapy dose per BSA and patient’s weight (Fig. 1). Patients who weighed over 100 kg tended to receive a lower dose of Flu per BSA than patients from the lower body weight cohort, however, the inverse dose-body weight correlation was not observed in Cyc dose (Supplementary Fig. S2). There was no significant difference in renal function between obese and non-obese patients (Table 1 and Supplementary Fig. S3)

**Table 1:** Characteristics and outcomes of patients with aggressive B-cell lymphoma treated with axicabtagene ciloleucel as stratified by obesity status.

Parameters	All Cohort *N* = 78	Non-Obese *N* = 59	Obese *N* = 19	*P*-Value^a^
Median age at the time of CAR T-cell infusion (years, range)	58.8 (26.8–76.5)	59.7 (26.8–76.5)	54.7 (30.1–70.1)	0.16
Age 60 years or older (%)	32 (41.0)	27 (45.8)	5 (26.3)	0.13
Male gender (%)	51 (65.4)	37 (62.7)	14 (73.7)	0.38
Advanced stage (3–4) (%)	74 (94.9)	57 (96.6)	17 (89.5)	0.25
Elevated lactate dehydrogenase (%)	53 (67.9)	41 (69.5)	12 (63.2)	0.61
ECOG performance status 2 or higher (%)	3 (3.8)	2 (3.4)	1 (5.3)	0.71
Median prior line of therapy before CAR T cells	3 (1–7)	3 (1–5)	3 (2–7)	0.21
Previous history of autologous stem cell transplant (%)	31 (39.7)	21 (35.6)	10 (52.6)	0.19
Patients with weight 100 kg or higher (%)	15 (19.2)	3 (5.1)	12 (63.2)	<0.001
Indication for CAR T-cell therapy (%):				0.29
Diffuse Large B Cell Lymphoma	51 (65.4)	38 (64.4)	13 (68.4)	
Transformed Follicular Lymphoma	18 (23.1)	12 (20.3)	6 (31.6)	
High Grade B Cell Lymphoma	8 (10.3)	8 (13.6)	0 (0)	
Prior history of central nervous system involvement (%)	10 (12.8)	9 (15.3)	1 (5.3)	0.44
Serum creatinine (mg/dL, range)	0.81 (0.54–2.64)	0.80 (0.56–2.1)	0.93 (0.54–2.64)	0.07
Creatinine clearance by MDRD equation (mL/min, range)	89.0 (222.9–140.6)	90.6 (22.9–130.5)	82.2 (26.0–140.6)	0.15
Creatinine clearance by CKG equation (mL/min, range)	103.4 (19.1–241.9)	97.8 (19.1–165.0)	135.8 (83.0–241.9)	0.001
Median duration from leukapheresis to CAR T infusion (day, range)	27 (20–356)	26 (20–356)	27 (24–33)	0.94
Median total delivered dose per body surface area of fludarabine (mg/m^2^, range)	89.1 (0–104.8)	89.2 (0–104.8)	88.7 (55.5–93.7)	0.36
Fludarabine dose reduction < 80% (%)	8 (11.0)	2 (3.7)	6 (31.6)	<0.001
Median total delivered dose per body surface area of cyclophosphamide (mg/m^2^, range)	1507 (1021–1660)	1512 (1021–1660)	1501 (1077–1525)	0.008
Cyclophosphamide dose reduction < 80% (%)	2 (2.7)	1 (1.9)	1 (5.3)	0.46
Median absolute lymphocyte count on the day of CAR-T infusion (× 10^9^/L, range)	0.02 (0.0–1.4)	0.02 (0.0–1.4)	0.02 (0.02–0.07)	1.00
CRS grade 2 or more severe (%)	35 (55.6)	26 (55.3)	9 (56.2)	0.95
Median time from CAR T infusion to onset of CRS (days, range)	3 (0–8)	4 (0–8)	3 (0–7)	0.11
Median duration of CRS (days, range)	5 (1–19)	5 (1–19)	7 (2–18)	0.10
ICANS grade 2 or more severe (%)	26 (65.0)	18 (64.3)	8 (66.7)	0.88
Median time from CAR T infusion to onset of ICANS (days, range)	5 (0–13)	5 (0–13)	6 (4–7)	0.96
Median duration of ICANS (days, range)	5 (1–20)	5 (1–20)	6 (2–17)	0.26
Receipt of tocilizumab for CRS treatment (%)	16 (20.5)	11 (18.6)	5 (26.3)	0.47
Receipt of systemic corticosteroid for CRS and ICANS treatment (%)	24 (30.8)	16 (27.1)	8 (42.1)	0.22
Median dose of systemic corticosteroid for CRS and ICANS treatment (mg/m^2^/day prednisone equivalent, range)	1.8 (0.6–21.2)	1.9 (0.7–21.2)	1.0 (0.6–10.2)	0.08
Median duration of systemic corticosteroid for CRS and ICANS treatment (days, range)	4 (1–15)	5 (1–15)	3 (1–11)	0.21
Median absolute lymphocyte count at 1-month post-CAR T-cell therapy (10^9^ cells/L, range)	0.46 (0–2.25)	0.48 (0–2.1)	0.38 (0.12–2.25)	0.43
Median absolute lymphocyte count at 3-months post-CAR T-cell therapy	0.44 (0.14–3.73)	0.49 (0.14–3.73)	0.31 (0.20–0.95)	0.10
Outcomes				
Best overall response rate (%)	56 (71.8)	43 (72.9)	13 (68.4)	0.71
1-year event free survival	34.6 (25.2–47.6)	35.8 (25.0–51.4)	30.7 (15.4–61.1)	0.60
1-year overall survival	65.5 (55.0–78.0)	59.4 (47.1–74.9)	83.9 (68.7–100.0)	0.18
1-year cumulative incidence of relapse	62.8 (51.5–74.2)	60.8 (47.4–74.2)	69.3 (46.8–91.8)	0.40
1-year cumulative incidence of non-relapse mortality	2.6 (0–6.1)	3.4 (0–8.0)	0 (0–0)	0.42
30-days cumulative incidence of CRS	83.3 (74.9–91.7)	83.1 (73.2–92.9)	84.2 (66.7–100)	0.36
30-days cumulative incidence of ICANS	51.3 (40.1–62.5)	47.5 (34–6–60.3)	63.2 (40.5–85.8)	0.23

*CAR* chimeric antigen receptor, *ECOG* Eastern Cooperative Oncology Group, *kg* kilogram, *CRS* cytokine release syndrome, *ICANS* immune effector cell-associated neurotoxicity syndrome, *MDRD* the modification of diet in renal disease.

^a^Comparison between groups for continuous variables by Wilcoxon rank-sum test, Comparison between groups for categorical variables by Chi-square test or Fisher Exact test, Comparison between groups for survival outcomes (survival, cumulative incidence) by Log-rank analysis.

**Fig. 1:**
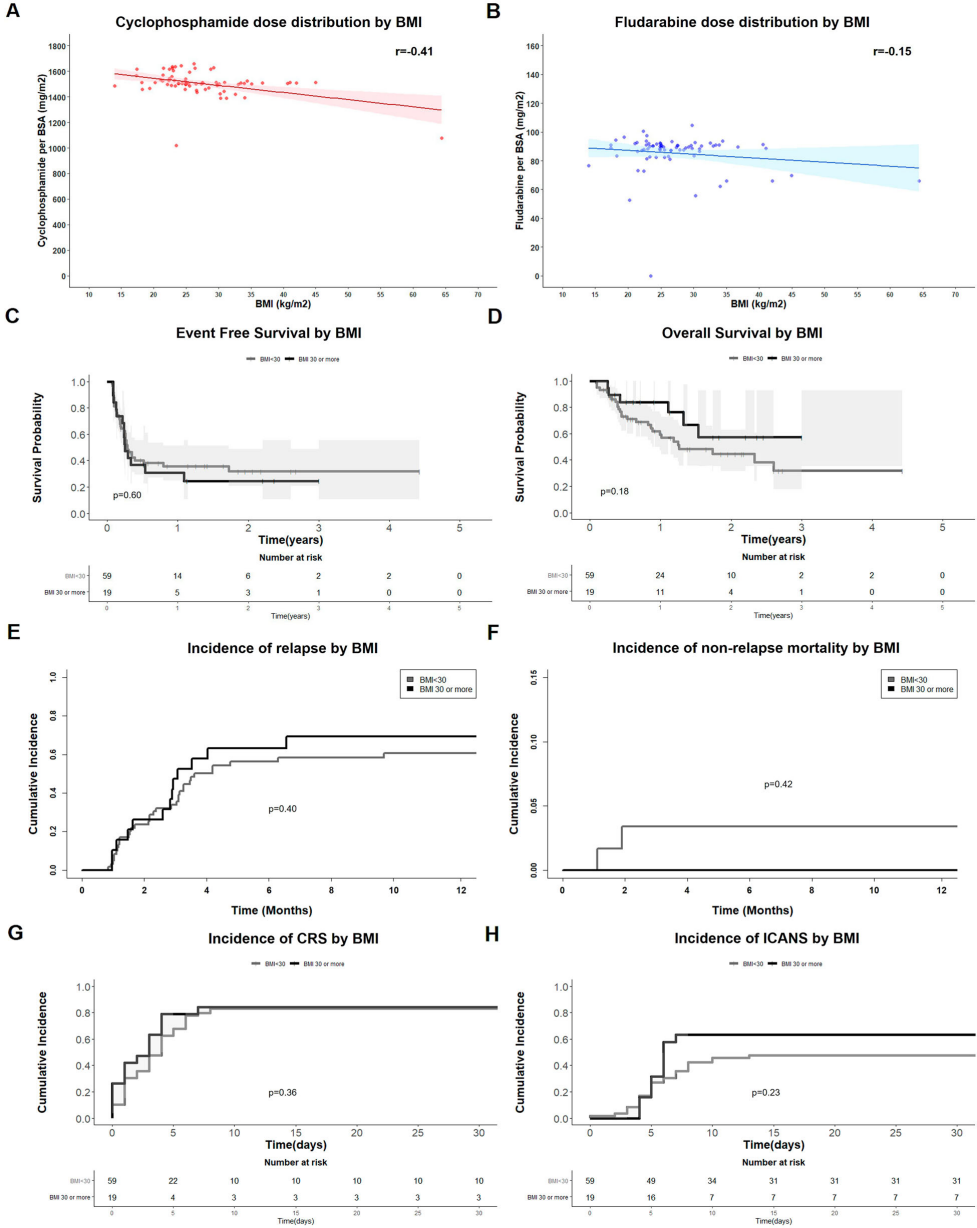
Correlation between body mass index (BMI) and administered dose of lymphodepletion chemotherapy and impacts of BMI on clinical outcomes. **A** Distribution of total delivered cyclophosphamide per body surface area and body mass index of 78 patients treated with axicabtagene ciloleucel. **B** Distribution of total delivered fludarabine per body surface area and body mass index of 78 patients treated with axicabtagene ciloleucel. **C** Event Free Survival after axicabtagene ciloleucel as stratified by obesity status. **D** Overall Survival after axicabtagene ciloleucel as stratified by obesity status. **E** Cumulative incidence of relapse after CAR T cell therapy as stratified by obesity status. **F** Cumulative incidence of non-relapse mortality as stratified by obesity status. **G** Cumulative incidence of CRS after axicabtagene ciloleucel as stratified by obesity status. **H** Cumulative incidence of ICANS after axicabtagene ciloleucel as stratified by obesity status.

Of the 77 patients evaluable for response, the best overall response rate (ORR) was 72.7% (CR 53.2%) and were comparable between obese (ORR 63.4%, CR 52.6%) and non-obese (ORR 74.1%, CR 53.4%) patients (*P* = 0.71). Among patients who attained partial response (PR) at month 1 post-CAR T-cell therapy (5 in obese group, 10 in non-obese group), the CR conversion rate was 40 and 80%, respectively. With a median follow-up of 15 months, 50 (64.9%) patients had relapsed, and 34 (43%) patients had died. The 1-year event free survival (EFS) and overall survival (OS) were 34.6% and 65.5%, respectively. Both 1-year EFS and OS were comparable between obese and non-obese groups (EFS 30.7% vs. 35.8%, *P* = 0.60 and OS 83.9 vs. 59.4%, *P* = 0.18) (Fig. 1). The cumulative incidence of relapse (CIR) and non-relapse mortality (NRM) in obese patients was not different compared to non-obese patients at 1-year (CIR 69.2% vs. 60.8%, *P* = 0.40 and NRM 0% vs 3.4%, *P* = 0.42) (Fig. 1). In cox proportional hazard regression analysis, the only factor associated with survival outcome was the total delivered dose of fludarabine <80% compared to standard per-protocol dose (HR 3.48, 95%CI 1.40–8.69, *P* = 0.007). The actual delivered Flu dose to the standard per-protocol dose ratio below 0.8 was associated with worse EFS and OS than those above 0.8; however, this association was not observed with Cyc. This finding supports the critical role of Flu in LD chemotherapy on outcomes in patients treated with CD19 CAR T-cell therapy. In addition, although our study did not show a direct impact of obesity including body weight on outcomes after CAR T-cell therapy, obesity might still influence the patterns of LD chemotherapy dosing, which may indirectly contribute to the success or failure of CAR T-cell therapy. No other factors were associated with survival outcomes after CAR T-cell therapy (Supplementary Table S1).

A total of 16 (84.2%) patients in the obese group developed cytokine release syndrome (CRS) compared to 49 (83.1%) in the non-obese group (*P* = 0.37, Fig. 1). The pattern of CRS, including time from CAR T infusion to CRS onset, maximal severity, and duration of CRS, were similar between the two groups. ICANS was observed in 12 (63.2%) and 28 (47.4%) patients in obese and non-obese group (*P* = 0.21, Fig. 1). There was no difference in the clinical course of ICANS in obese compared to non-obese patients. There was also no difference in the inflammatory markers CRP and ferritin during CAR T-cell therapy (baseline, peak level and interval trend of CRP, ferritin between obese and non-obese patients (Supplementary Fig. S4). There was no difference observed in hematologic recovery, including hemoglobin, platelet, neutrophil count, and B cell counts (Supplementary Fig. S5).

In conclusion, our study demonstrated no association between obesity and immune-mediated toxicities, kinetics of B-cell aplasia, CAR T efficacy, or survival. In contrast to the impact of obesity on HSCT and ICB therapy [5], our findings, may indicate the differential interaction between obesity and various immunotherapeutic modalities. However, the power of our study was limited by the small number of obese patients. In addition, selection bias upon physician’s discretion on patient’s treatment assignment could lead to unbalanced distribution between groups, which may potentially affect the outcomes of patients in our study. Although we did not observe a correlation of obesity with outcomes, it might influence LD chemotherapy dosing patterns. Historically, obesity has been an important factor influencing the chemotherapy dosing in oncology practice and conditioning regimens in HSCT. Several studies have examined the effect of pharmacokinetics (PK) of different conditioning chemotherapy regimens including Flu on various clinical endpoints after HSCT [10, 11]. Several early phase studies have shown that Flu, when given as LD chemotherapy, improved CAR T-cell expansion and outcomes after CAR T-cell therapy compared to single-agent Cyc [12]. Although there is currently no PK-directed data about the effect of Flu on toxicities and outcomes after CAR T-cell therapy, physicians should be mindful about potential undesirable consequence on outcomes from LD chemotherapy dose reduction especially in patients with extreme body weight [13]. Whether obesity impacts toxicities and outcomes in patients treated with CAR T-cell therapy, further studies from larger multi-centered cohorts including correlative studies on CAR T-cell kinetics are warranted to establish the significance of obesity on the outcome after CAR T cells.

## Supplementary information

Supplementary material
