# Supplementary material for: The impact of obesity and body weight on the outcome of patients with relapsed/refractory large B-cell lymphoma treated with axicabtagene ciloleucel
Source: Blood Cancer J. 2021 Jul 1;11(7):124. doi: 10.1038/s41408-021-00515-2 (PMC8249448; doi:10.1038/s41408-021-00515-2)
Supplement: Supplementary file 1 — Supplementary material [file 41408_2021_515_MOESM1_ESM.docx]

**Supplementary Material**

**Supplementary Figure S1:** Distribution and correlation of weight and height at the time of LD chemotherapy A) The whole patient cohort (N=78) B) Stratified by obesity status


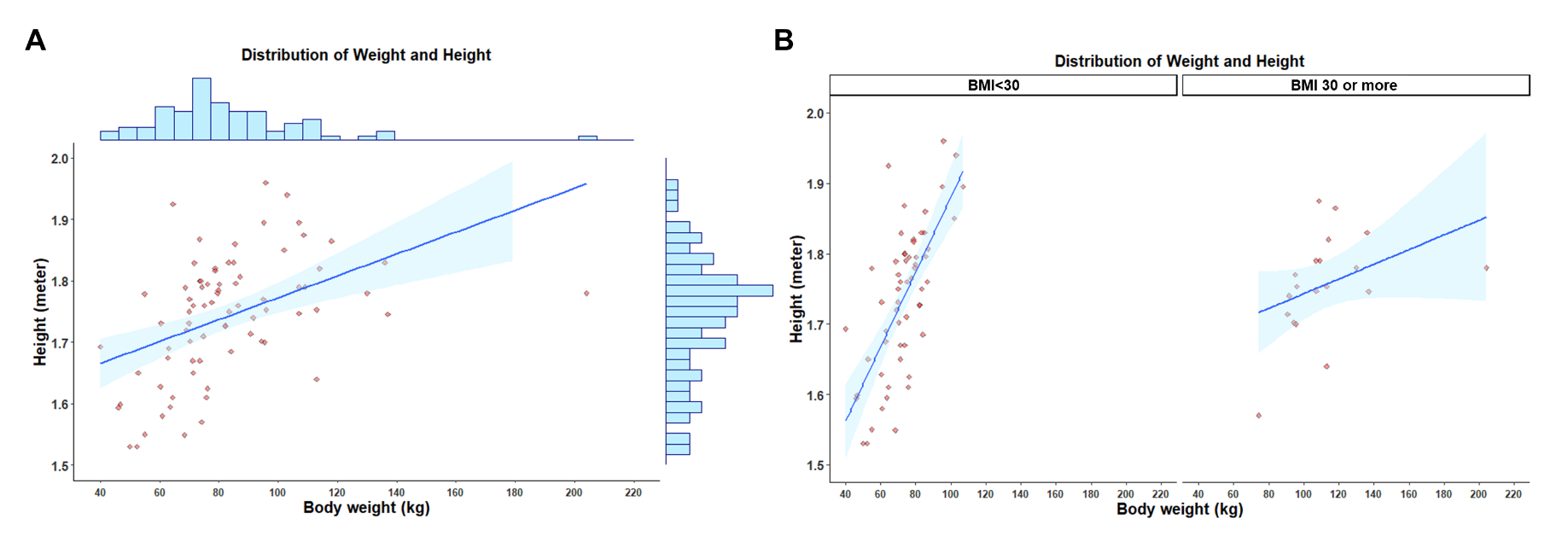


**Supplementary Figure S2:** Distribution and correlation of weight and delivered LD chemotherapy dose per body surface area (mg/m^2^) grouping by body weight at the time of LD chemotherapy initiation excluding 1 outlier patient A) Cyclophosphamide B) Fludarabine


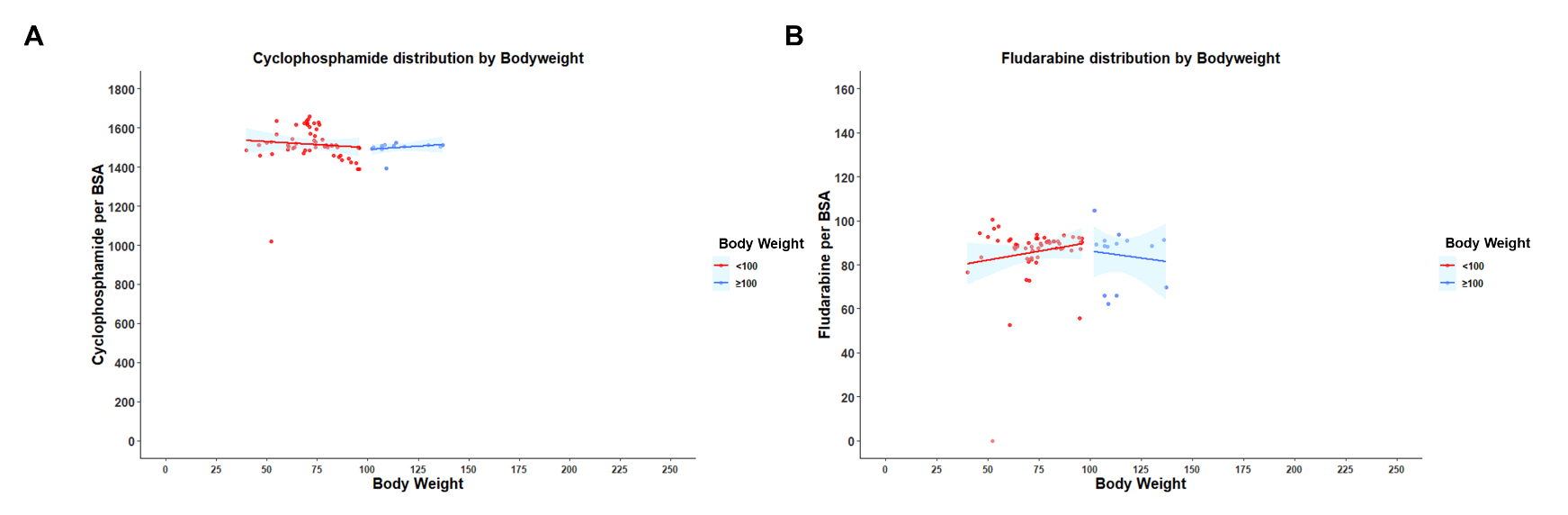


**Supplementary Figure S3:** Correlation between age, body weight, BMI, creatinine, creatinine clearance (by Cockcroft-Gault and the Modification of Diet in Renal Disease study equations)


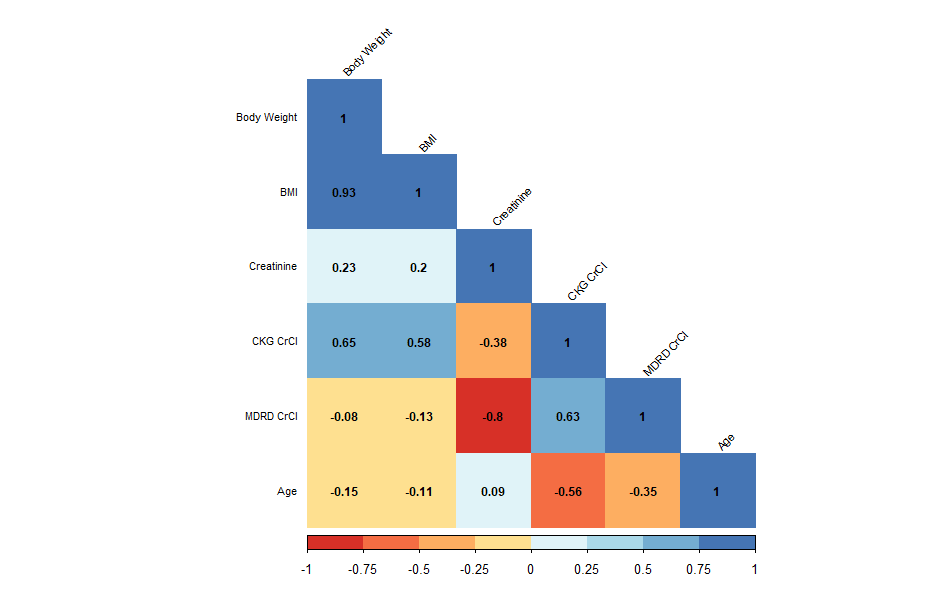


**Supplementary Figure S4:** Kinetics of CRP and Ferritin at baseline pre-LD chemotherapy, peak level and incremental change from baseline to peak level as stratified by obesity status A) CRP B) Ferritin


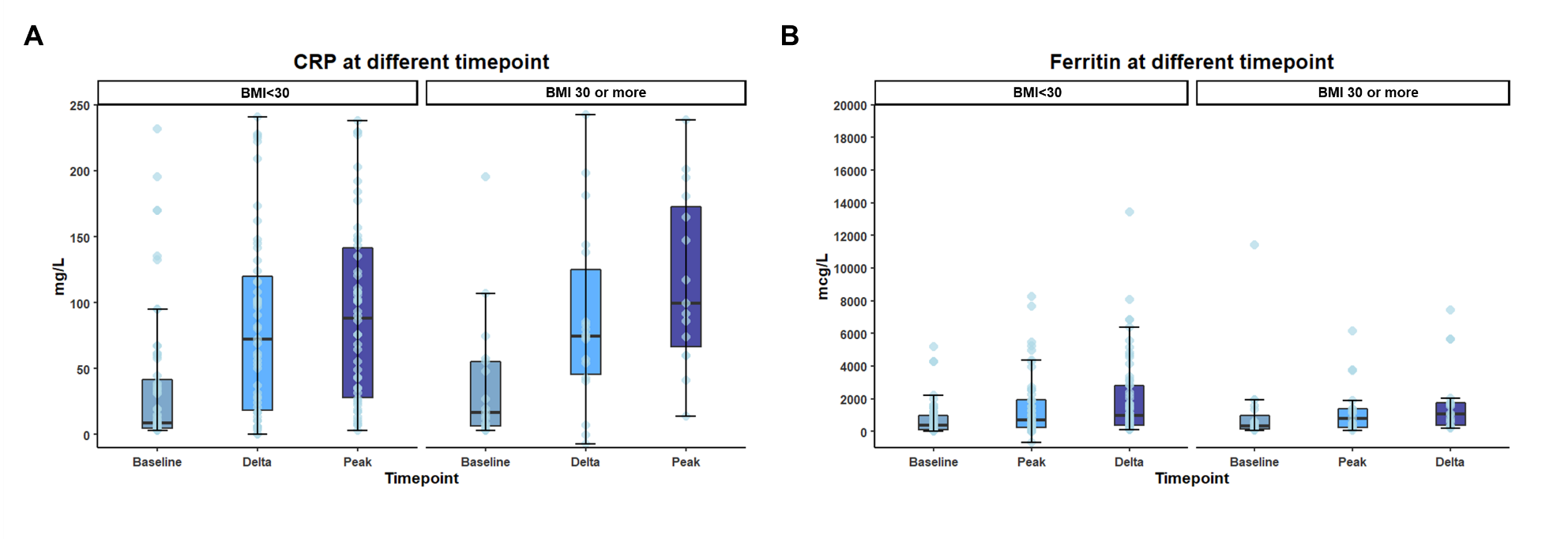


**Supplementary Figure S5:** Hematologic Recovery at 1 months and 3 months post-CAR T cell therapy stratified by obesity status A) Hb at 1 month B) Hb at 3 months C) WBC at 1 month D) WBC at 3 months E) ANC at 1 month F) ANC at 3 months G) ALC at 1 month H) ALC at 3 months I) Platelet at 1 month J) Platelet at 3 months. There was no significant difference between median Hb, WBC, ANC, ALC, platelet between obese and non-obese at either 1- or 3-months post-CAR T cell therapy.


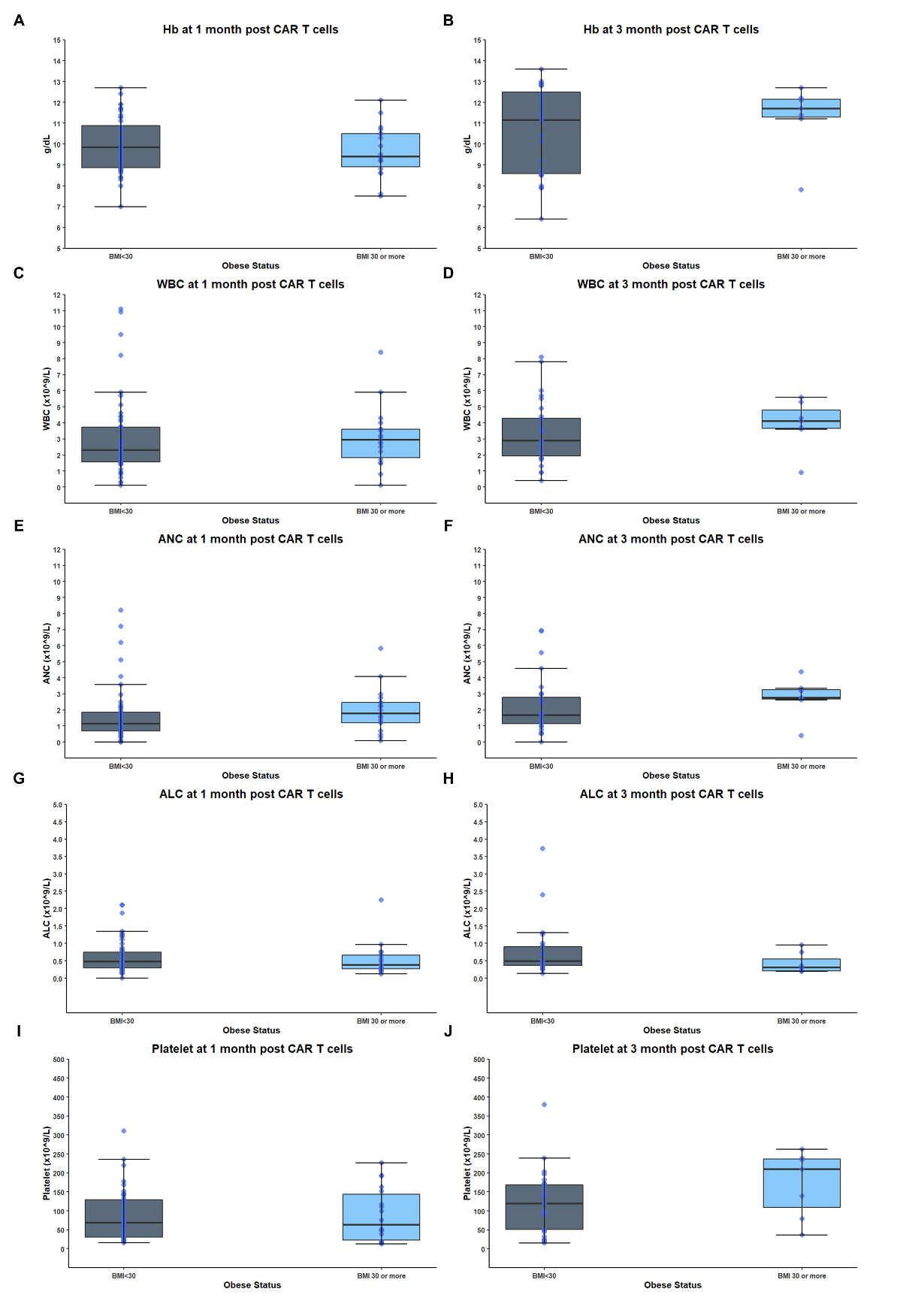


**Supplementary Table S1:** Cox Proportional Hazard Analysis of prognostic factors for overall survival after CAR T cell therapy

|  | **Hazard Ratio (95%CI)** | ***P*-value** |
| --- | --- | --- |
| **Age (≥60 years vs. <60 years)** | 1.79 (0.91-3.53) | 0.09 |
| **Gender (Male vs. Female)** | 0.84 (0.41-1.71) | 0.63 |
| **Stage (Advanced vs. Early)** | 1.87 (0.57-6.12) | 0.30 |
| **LDH (Elevated vs. Normal)** | 1.51 (0.73-3.10) | 0.26 |
| **ECOG Performance status (≥2 vs. 0-1)** | 1.72 (0.23-13.0) | 0.60 |
| **Line of therapy (≥3 vs. <3)** | 1.28 (0.56-2.94) | 0.56 |
| **Prior history of ASCT (Yes vs. No)** | 0.89 (0.44-1.77) | 0.73 |
| **Obesity (BMI≥30 vs. BMI<30)** | 0.55 (0.23-1.34) | 0.19 |
| **Body weight (≥100 vs. <100)** | 0.79 (0.32-1.91) | 0.60 |
| **Delivered to standard dose ratio of fludarabine (<80% vs. ≥80%)** | 3.48 (1.40-8.69) | **0.007** |
| **Delivered to standard dose ratio of cyclophosphamide (<80% vs. ≥80%)** | 4.97 (0.62-39.55) | 0.13 |
| **CRS (Grade≥2 vs. Grade 0-1)** | 1.50 (0.69-3.29) | 0.30 |
| **ICANS (Grade≥2 vs. Grade 0-1)** | 1.01 (0.37-2.74) | 0.98 |
